# Gasoline cars produce more carbonaceous particulate matter than modern filter-equipped diesel cars

**DOI:** 10.1038/s41598-017-03714-9

**Published:** 2017-07-13

**Authors:** S. M. Platt, I. El Haddad, S. M. Pieber, A. A. Zardini, R. Suarez-Bertoa, M. Clairotte, K. R. Daellenbach, R.-J. Huang, J. G. Slowik, S. Hellebust, B. Temime-Roussel, N. Marchand, J. de Gouw, J. L. Jimenez, P. L. Hayes, A. L. Robinson, U. Baltensperger, C. Astorga, A. S. H. Prévôt

**Affiliations:** 1 Paul Scherrer Institute, Laboratory of Atmospheric Chemistry, CH-5232 Villigen, Switzerland; 2 NILU-Norwegian Institute for Air Research, PO Box 100, 2027 Kjeller, Norway; 3 European Commission Joint Research Centre, Directorate for Energy, Transport and Climate, Sustainable Transport Unit, 21027 Ispra, (VA) Italy; 4 Aix Marseille Univ, CNRS, LCE, Marseille, France; 5 NOAA Earth System Research Laboratory, Boulder, CO USA; 6 CIRES, University of Colorado, Boulder, CO USA; 7 Department of Chemistry, University of Colorado, Boulder, CO USA; 8 Département de Chimie, Université de Montréal, Montréal, Québec Canada; 9 Center for Atmospheric Particle Studies, Carnegie Mellon University, Pittsburgh, PA 15213 USA; 10 Key Laboratory of Aerosol Chemistry & Physics, State Key Laboratory of Loess and Quaternary Geology, Institute of Earth Environment, Chinese Academy of Sciences, Xi’an, 710061 China; 11 Central Statistics Office, Cork, Ireland

## Abstract

Carbonaceous particulate matter (PM), comprising black carbon (BC), primary organic aerosol (POA) and secondary organic aerosol (SOA, from atmospheric aging of precursors), is a highly toxic vehicle exhaust component. Therefore, understanding vehicle pollution requires knowledge of both primary emissions, and how these emissions age in the atmosphere. We provide a systematic examination of carbonaceous PM emissions and parameterisation of SOA formation from modern diesel and gasoline cars at different temperatures (22, −7 °C) during controlled laboratory experiments. Carbonaceous PM emission and SOA formation is markedly higher from gasoline than diesel particle filter (DPF) and catalyst-equipped diesel cars, more so at −7 °C, contrasting with nitrogen oxides (NO_X_). Higher SOA formation from gasoline cars and primary emission reductions for diesels implies gasoline cars will increasingly dominate vehicular total carbonaceous PM, though older non-DPF-equipped diesels will continue to dominate the primary fraction for some time. Supported by state-of-the-art source apportionment of ambient fossil fuel derived PM, our results show that whether gasoline or diesel cars are more polluting depends on the pollutant in question, i.e. that diesel cars are not necessarily worse polluters than gasoline cars.

## Introduction

Most carbonaceous PM from passenger cars is SOA^1–4^, known to contain harmful reactive oxygen species^5^ and damage lung tissue^6^. However, despite extensive investigation of diesel car pollution^7^, the relative contributions of diesel and gasoline cars to ambient SOA remains unquantified. While current EU-average passenger car fleet PM emission factors are much higher for diesel than gasoline^8^, these values are skewed by older vehicles and do not reflect the impacts of the latest generation of after-treatment devices. Diesel passenger cars sold today in the EU/US have diesel particle filters (DPF)^9^, a wall-flow filter often coated or associated with an oxidation catalyst. Therefore, in order to assess the relative merits of different engine technologies and mitigate vehicle pollution, knowledge of the primary emissions and SOA formation from modern passenger cars equipped with the newest after-treatment technologies is required.

While previous studies have investigated carbonaceous aerosol emission/formation from vehicles via a bottom-up approach (laboratory quantification of tailpipe emissions)^1, 10^ or top-down source apportionment approaches^11^, here we combine both. We quantify carbonaceous PM from modern passenger cars (Euro 5 gasoline and DPF-equipped diesel, see Supplementary Information (SI) Table S1 for pollutant limit values). We then constrain our measurements using state-of-the-art source apportionment investigations, providing a comprehensive assessment of the current state and future trends in gasoline and diesel vehicular pollution. Further, we present laboratory measurements of gasoline car SOA formation at low temperatures (−7 °C), in addition to measurements at 22 °C. Low temperatures significantly increase vehicle total hydrocarbon (THC) emission^12, 13^, including SOA precursors, and favour condensation of gases to the aerosol phase^14^. With these measurements, we parameterise vehicular SOA formation, accounting for the full range of relevant ambient temperatures, background OA concentrations, and extent of atmospheric aging. This parametrisation method is new and, critically, does not require direct knowledge of precursor composition (which typically includes unknown and/or unquantifiable gases).

**Figure 1:**
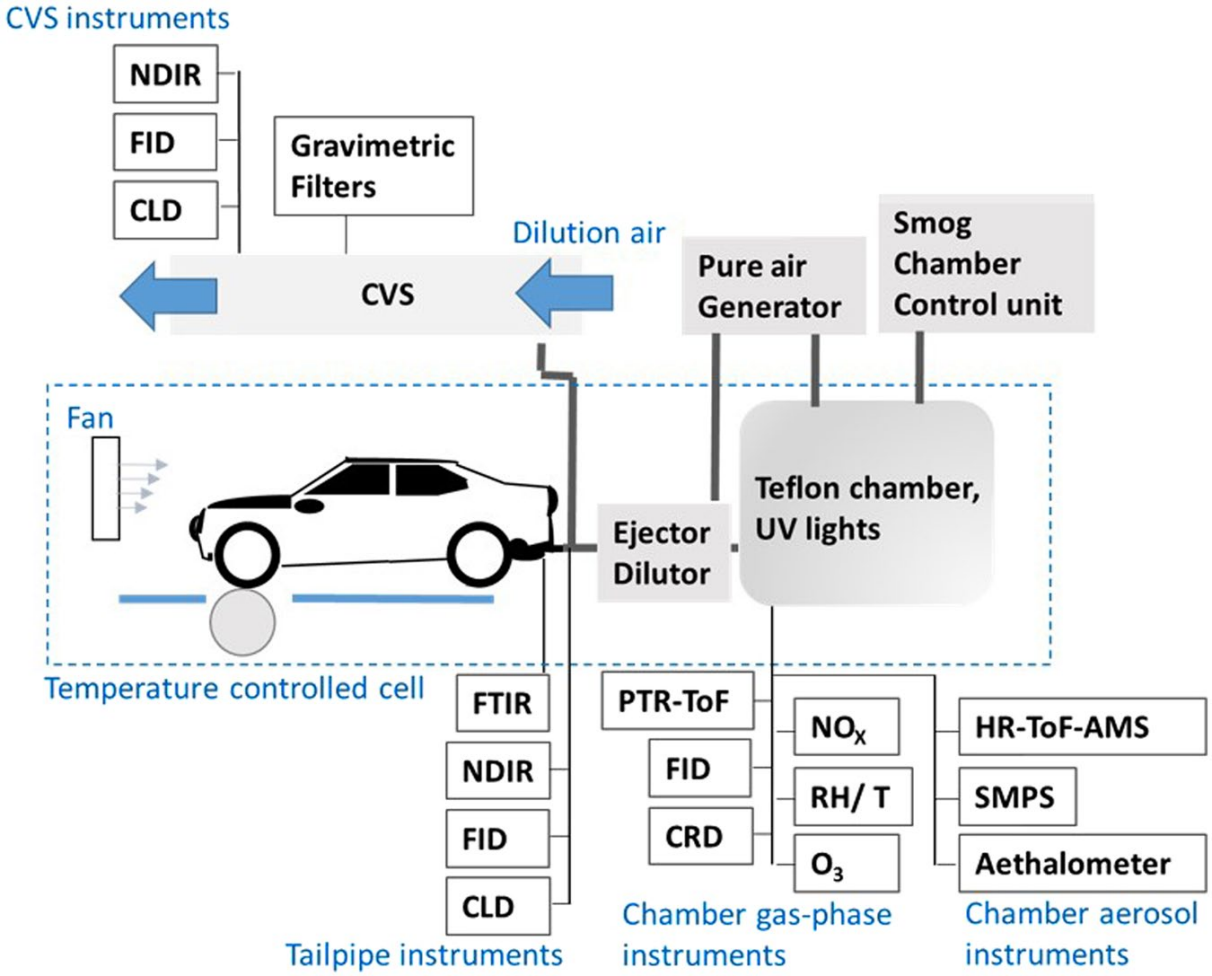
Schematic (not to scale) of the experimental set up. The test vehicle and smog chamber were operated inside the temperature controlled test cell with instrumentation outside. During testing, instruments were operated at the tailpipe and from a constant volume sampler (CVS), while a small fraction of emission were sampled into the smog chamber via a heated ejector dilutor (total dilution factor ~150). Tailpipe instrumentation included a Fourier-transformed infrared spectrometer (FTIR), non-dispersive infrared sensor (NDIR), flame ionisation detector (FID), and a chemiluminescence detector (CLD). At the CVS were NDIR, CLD, and FID instruments as well as a heated gravimetric filter sampler. After testing, primary emissions were investigated and then aged in the smog chamber under UV lights. Gas phase instruments were a proton transfer reaction time-of-flight mass spectrometer (PTR-ToF-MS), FID, cavity ring-down spectrometer, nitrogen oxide (NO_X_) detectors, an ozone (O_3_) monitor and relative humidity and temperature sensors (RH/T). Aerosol instrumentation was a high-resolution time-of-flight aerosol mass spectrometer (HR-ToF-AMS), a scanning mobility particle sizer (SMPS) and an aethalometer. Table S2 lists all chamber instruments in detail while Platt *et al*.^4^ give a fuller description of this set-up.

**Figure 2:**
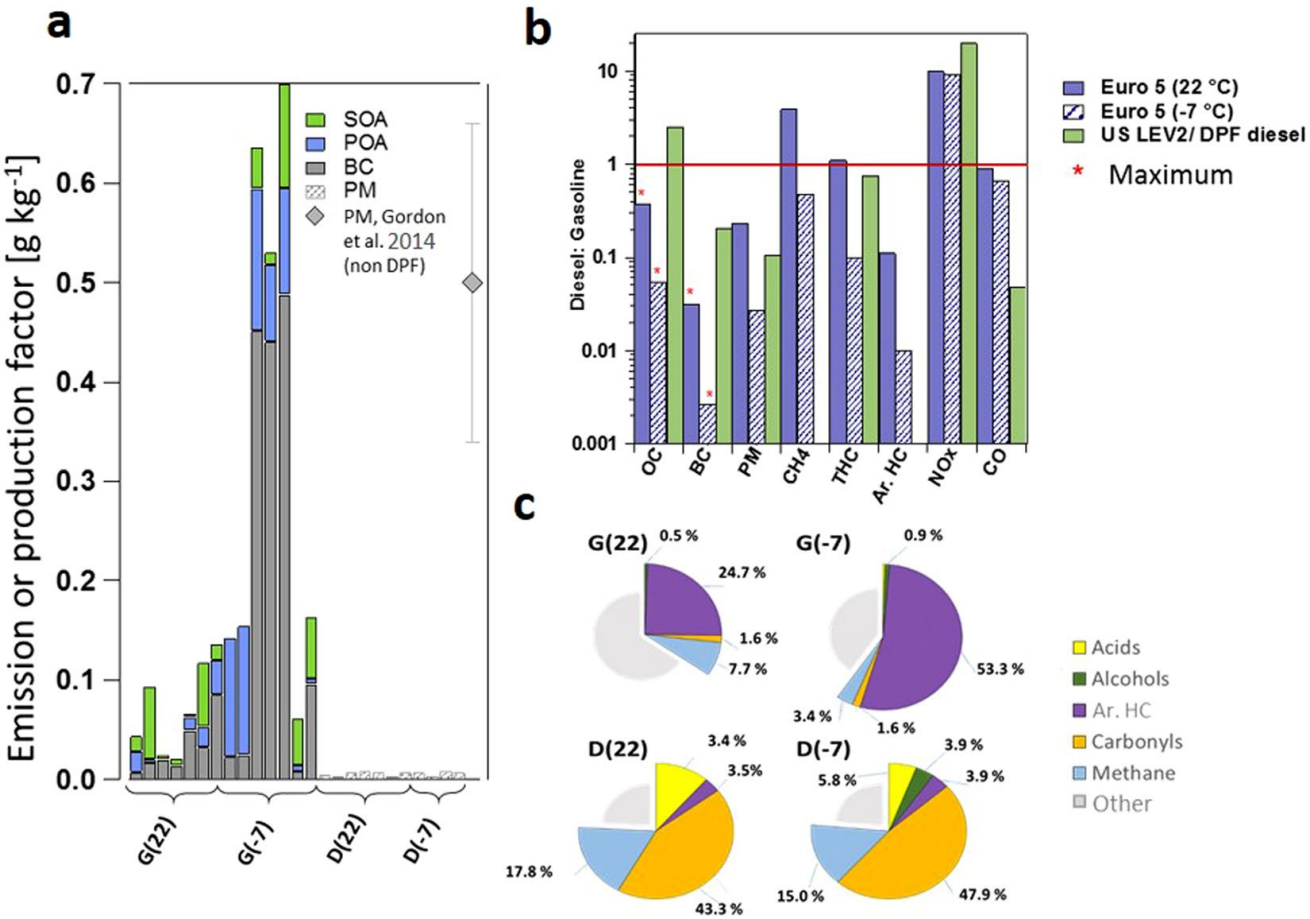
Carbonaceous emissions/secondary organic aerosol formation from modern diesel and gasoline passenger cars. (**a**) Aerosol emission factors (g kg^−1^ fuel) measured in this study. SOA is at OH exposure = 10^7^ molec. cm^−3^ h. Diesel vehicles did not produce measurable OA so gravimetric PM is given. For comparison the average and standard deviation of PM emission factors from non-DPF medium duty diesels from Gordon *et al*. 2013 ref. 3, is shown. (**b**) Average ratio of diesel/gasoline emission factors for OA, BC, PM, methane (CH_4_), total hydrocarbon (THC), aromatic hydrocarbons (Ar. HC), nitrogen oxides (NO_X_) and carbon monoxide (CO) at 22 °C and −7 °C. Euro 5 values are obtained using the NEDC, while US LEV2 and DPF-equipped vehicles use the US unified driving cycle, UC^3, 21^. While a true ratio cannot be calculated for OC and BC, a maximum can be, based on detection limits, highlighted by the red asterisks (*). In contrast to CO and especially NO_X_, PM and hydrocarbon emissions from gasoline cars are higher than from diesel cars. (**c**) Averaged THC-normalised exhaust composition (temperature in parentheses) in the smog chamber with uncharacterised emissions in grey. The single largest fraction of gasoline THC consists of methyl benzenes present in the fuel, while the rest likely consists of surviving linear/branched, saturated/unsaturated hydrocarbons. Meanwhile, diesel emissions mainly comprise pyrolysis products, including small carbonyls (formaldehyde, acetaldehyde) and carboxylic acids (formic, acetic), which are not efficient SOA precursors. Supporting material related to Fig. 2 available in SI Tables S3–7, SI.

**Figure 3:**
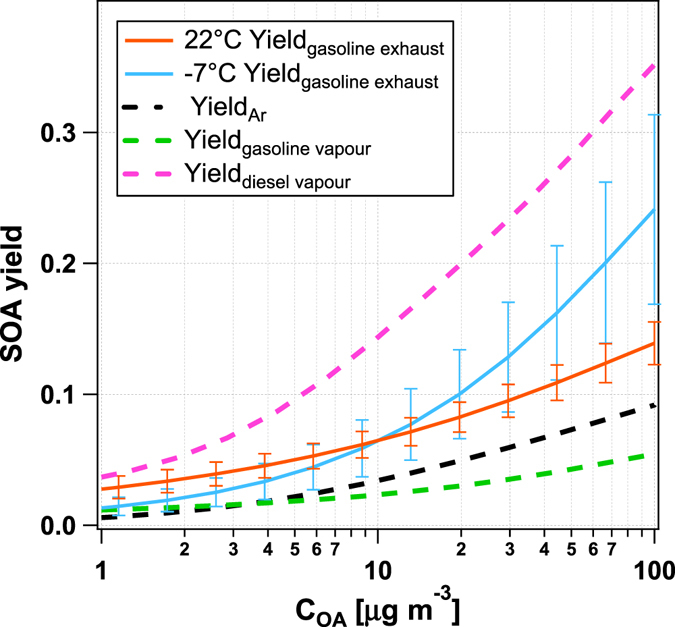
Secondary organic aerosol yields from Euro 5 gasoline car emissions. Modelled secondary organic aerosol (SOA) yields as a function of suspended organic aerosol (C_OA_) concentration from three Euro 5 gasoline cars at 22 and −7 °C (orange and blue, respectively). Error bars represent one standard deviation. Also shown: SOA yields from light aromatic hydrocarbons (Yield_Ar_), and diesel and gasoline vapours (Yield_diesel vapour_, Yield_gasoline vapour_), from Jathar *et al*.^38^. The comparison clearly highlights that SOA yields from gasoline emissions are higher than those based on the fuel aromatic content or expected from gasoline vapour, suggesting the presence of unidentified SOA precursors in the exhaust. Meanwhile, DPF-diesel emissions did not produce any measureable SOA, in contrast to the oxidation of diesel vapours. Taken together both of these observations indicate that SOA yields from gasoline and diesel emissions cannot be predicted based on the yields of fuel vapours. Note that the gasoline car SOA yields are not corrected for vapour phase wall losses (see Methods), for the purposes of intercomparison with previous studies.

Figure 1 shows the experimental set-up used in this study, while details of instrumentation, the test fleet, and smog chamber experimental conditions are given in SI Tables S2–5. Figure 2a shows a comparison of emission factors (EF, g carbon (C) kg^−1^ fuel) of carbonaceous aerosol and SOA formation during the New European Driving Cycle (NEDC, SI Fig. S1, from in-use Euro 5 gasoline and DPF-equipped-diesel passenger cars, measured in a smog chamber^4^. DPF-equipped-diesel car EFs are orders of magnitudes below inventory values (0.68–2.64 g kg^−1^ fuel)^8^ and far below those reported for older non-DPF-equipped passenger cars^3, 15^, while gasoline cars are comparable to inventory values (0.01–0.04 g kg^−1^ fuel). The gasoline cars emitted on average 10 times more carbonaceous aerosol at 22 °C and 62 times more at −7 °C compared to diesel cars, mainly due to substantially higher BC, with EF at −7 °C comparable to those from old diesel. Low temperatures dramatically increased primary emissions and secondary carbonaceous aerosol formation from the gasoline cars but not diesel. Strikingly, for one gasoline car, BC emissions were at least 400 times higher than from the diesels (comparing to the detection limit). The increase in the emissions at lower temperatures is related to a more pronounced cold start effect likely resulting from (1) a poorer combustion efficiency following energy loss to cold engine surfaces and increased friction due to lubricants being too viscous at low temperatures; and of (2) an extended delay before catalyst light-off. These factors tend to be more important in gasoline vehicles than diesel vehicles^16, 17^. The diesel cars emitted 10 times more NO_X_ at both temperatures. Real world vehicle emissions of NO_X_ tend to differ from laboratory results^18, 19^. Importantly however, such biases are not expected for other pollutants, e.g. THC^20^. If SOA were to be added to the primary emissions, the inventory values for the gasoline cars would be exceeded by far: Emissions from new gasoline cars (both EU and US) produce up to 6.5 times more SOA than POA after 5–10 hours of atmospheric aging^2^. Meanwhile, the new diesel cars produced no detectable SOA, contrasting with old diesels, for which SOA production is about equal to emitted POA^3^. The absence of observed SOA from the DPF-diesels is explained by the chemical composition of the THC emissions; while emissions from non-DPF-diesels chemically resemble diesel vapours^21^, DPF-equipped-diesel exhaust comprises a large fraction (>70%) of short-chain oxygenated compounds, mainly formaldehyde and acetaldehyde, which are much less efficient SOA precursors than the aromatic species found in gasoline emissions (Fig. 3c). These results challenge the existing paradigm that diesel cars are associated, in general, with far higher PM emission rates^8^, reflecting the effectiveness of recent, Euro 5–6, diesel after-treatments such as DPFs combined with diesel oxidation catalysts. Furthermore, while Gordon *et al*.^3^ report primary PM emissions (g kg^−1^ fuel) during DPF regeneration (burn-off of accumulated PM) similar to those during regular driving of a non-DPF vehicle, DPF regeneration is activated infrequently (every few hundred km), lasts around one minute, and is more likely to occur at high speed (more likely outside densely populated areas). Thus, the net result of regeneration is unlikely to significantly diminish DPF efficacy. For all emission factors and SOA production factors for the different tests (including in g km^−1^), please see SI Tables S6–7.

**Figure 4:**
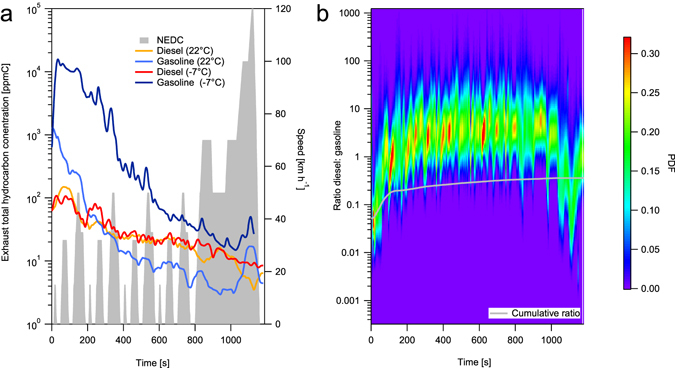
Time resolved exhaust total hydrocarbon (THC) concentrations from Euro 5 gasoline (number vehicles, n = 11) and diesel (n = 6) passenger cars. (**a**) Median time-resolved THC exhaust concentrations in the diesel and gasoline passenger car exhausts during the NEDC driving cycle, at −7 and 22 °C (target speed, grey shaded region) (**b**) Ratio of the distributions whose medians are given in A, shown as a probability density function (PDF, colour scale), for the 22 °C case. While the ratio of diesel to gasoline exhaust THC is broadly distributed (>factor 10) and variable, particularly during initial operations at higher speed, the difference is still statistically significant. The grey line gives the time-integrated median of the distribution (i.e. from the test start to the denoted time, lower than the time-resolved ratio for much of the cycle due to the high absolute concentrations and low gasoline:diesel THC during the cold start, and below unity, indicating that total gasoline exhaust THC emissions are higher). Note the logarithmic scale in both panels. While THC emissions from diesel cars are up to one order of magnitude higher than gasoline emissions during almost the entire driving period, gasoline emissions are up to two orders of magnitudes higher when the catalyst is cold. Because of the cold start effect, integrated emissions from gasoline cars exceed diesel, even after a journey of several kilometres (~14 km). This effect is more pronounced at −7 °C (SI Fig. S3).

Figure 3 shows that SOA yields (ΔSOA/ΔTHC) for gasoline passenger cars are higher at −7 compared to 22 °C and exceed those of both raw gasoline and pure single-ring aromatic hydrocarbons. These yields were determined using a new methodology, as described in the SI. We also estimated an effective enthalpy of vaporisation (encompassing the effect of non-ideal mixing) of 19 kJ mol^−1^, and a change in yield of 2 ± 0.4% K^−1^. For SOA yields as a function of temperature and suspended OA concentrations, please see SI Fig. S6. Since gasoline cars produced SOA whereas the DPF-equipped diesels did not, the expectation is that gasoline passenger cars should dominate urban vehicular SOA^11, 22–24^. However not all studies agree, for example Gentner *et al*.^25^ suggest that diesel vehicles produce more SOA since 1) most THC along a highway comes from diesels and 2) exhaust THC is similar to vaporised fuel, for which diesel SOA yields are higher (Fig. 3). One explanation for this may be that assumption 1 is invalid for newer vehicles, since the THC composition from new diesels comprises a large fraction of low molecular weight carbonyls not present in raw diesel fuel (Fig. 2). Another explanation may be that roadside measurements do not capture the large cold-start related emissions of THC from gasolines likely occurring before reaching major highways (Fig. 4). Our results show that emissions from vehicles are sensitive to sampling location, fleet age and ambient temperature, implying that regional studies representing an aggregate of emissions are likely to produce better estimates of the relative importance of diesel vs. gasoline to ambient SOA than roadside studies at one location.

**Figure 5:**
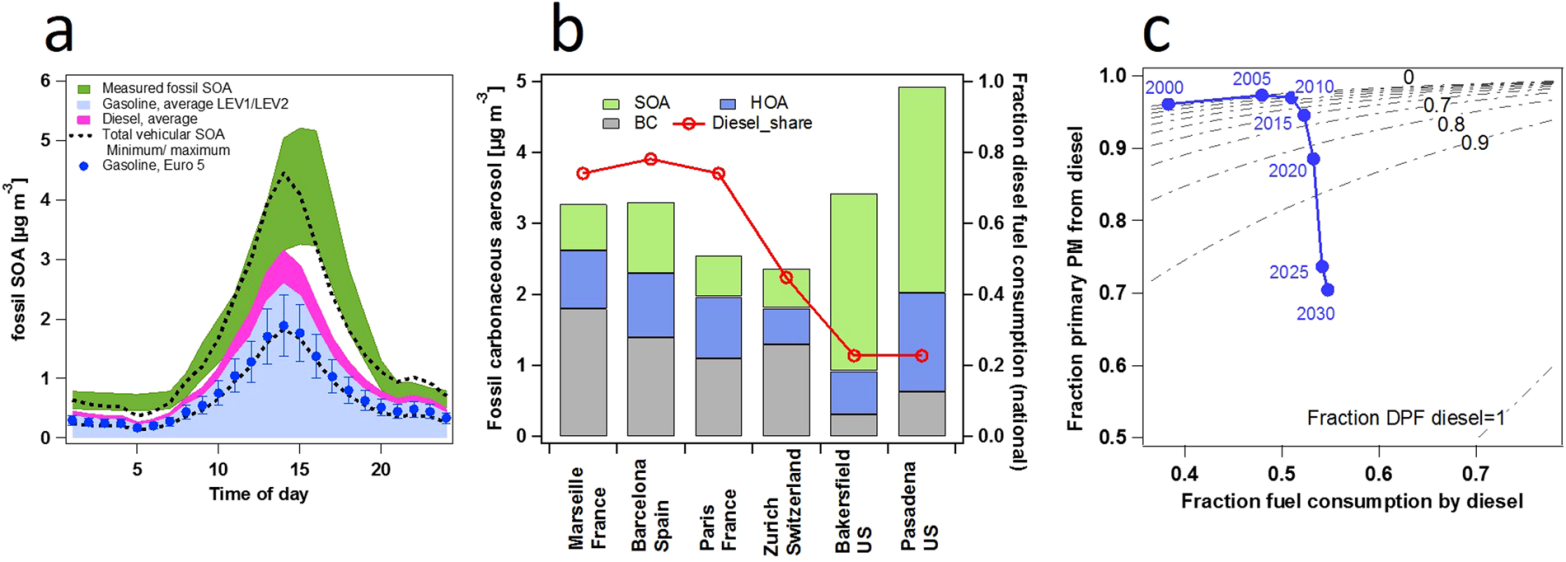
Estimates of the contribution of diesel and gasoline passenger cars to ambient urban PM and carbonaceous aerosol. (**a**) Contributions of gasoline (blue, shaded) and diesel cars (pink, shaded) to fossil SOA (green, shaded) in the L.A. basin, incorporating ambient OH exposure measurements, chamber SOA yields for LEV1/LEV2 vehicles, and ambient tracer concentrations (see SI for methodology). Maxima-minima for total vehicular SOA given by black dashed lines. Blue markers show an estimate from Euro 5 yields for comparison. Uncertainties in OH exposures are unaccounted for, possibly explaining the time shift between model/observed peak concentrations. For the measured fossil SOA, the range is determined by propagating the best estimate of the errors in the determination of SOA from Hayes *et al*.^26^ and those related in the determination of SOA fossil fraction from ^14^C measurements in Zotter *et al*.^27^. (**b**) Black carbon (BC), hydrocarbon-like organic aerosol (HOA) and fossil-fuel related (f) SOA and national share of diesel on-road vehicles (all types) according to the GAINS model^39^. The composition of the fossil carbonaceous matter in Europe and the US is clearly different, with the dominance of BC from diesel emissions in Europe and of fossil SOA from gasoline emissions in the US consistent with bottom-up calculations. (**c**) Fractional contribution of diesel passenger cars to primary vehicular PM (gasoline + diesel) as a function of total passenger car fuel consumption and fraction of DPF diesels, at 22 °C. Dashed lines show the effect of the DPF fraction on diesel vehicles’ share of PM emissions at any given fraction fuel consumption. A projection for the EU is shown with data on the fraction of Euro 5 diesel from the TREMOVE model^30^. For the current fleet, primary PM from gasoline cars would only exceed those from diesel when 97% of diesels are DPF-equipped. SOA would exhibit a similar, but less pronounced trend. Additional data are shown in SI Fig. S5 and SI Table S11.

We further show that gasoline vehicle SOA can comprise a large, sometimes dominant fraction of vehicle SOA by modelling ambient SOA from gasoline and old diesel cars in the L.A. region (Fig. 5a, see also SI materials) and comparing the result to observed ambient fossil fuel-related SOA (*f*_SOA_)^24, 26, 27^. We use toluene as a tracer for gasoline car emissions and the yields from our smog chamber experiments, since Borbon *et al*.^28^ demonstrate that traffic is the predominant source of toluene in L.A. based on the toluene to CO ratio, while our own experiments show minimal toluene emission from diesel. Furthermore, Baker *et al*.^6, 29^ show that during the CALNEX campaign, and using emission inventory data, non-vehicular point sources account for 12% and 6% of benzene + toluene + xylenes (BTEX) in Bakersfield and Pasadena, respectively.

Modelled SOA fits within uncertainties the measured *f*_SOA_ assuming yields from LEV1/LEV2/Euro 5, while gasoline emissions alone may explain up to 82% of observed *f*_SOA_. Meanwhile, the estimated contribution from diesel is significantly lower. While we do not achieve closure with the ambient data, the important information is the split between *f*_SOA_ from gasoline and diesel vehicles showing that gasoline is the larger fraction. This conclusion agrees with ambient observations of fossil fuel-related aerosol in European cities and the US (Fig. 5b). POA and BC are the largest fraction of carbonaceous PM in Europe, consistent with a higher share of diesel cars. At these locations, *f*_SOA_ is likely mainly from non-DPF diesel emissions, assuming it is approximately equal to diesel POA emissions^12^. In the US, where cars are almost exclusively gasoline powered, SOA is the dominant fraction of carbonaceous PM, as would be expected from the bottom-up estimations in Fig. 5a. Therefore, in combination, the laboratory SOA yields and ambient measurements suggest a large and likely increasingly dominant fraction of carbonaceous aerosol is from gasoline passenger cars in the US, while in Europe, old non-DPF diesels still dominate the carbonaceous aerosol burden.

We emphasise that since PM emissions are much higher from diesel cars without a DPF, diesel passenger car carbonaceous emissions will continue to be important for some time. Figure 5c shows an estimation of the impact of a gradual phase-in of DPFs on the fraction of primary vehicular PM from diesel^30^. A projection for the EU, shown in blue, suggests an initial increase in the share of primary PM from diesel vehicles, followed by a decrease as DPFs become widespread. Adoption of electric vehicles does not alter the conclusions, as they do not affect the relative contribution from either gasoline or diesel passenger cars. Results show that in the presence of only 3% of non-DPF diesel cars, diesel cars would still dominate car primary carbonaceous emissions, in the EU in warm conditions. Meanwhile, during winter, gasolines may already dominate passenger car carbonaceous PM at present.

Higher carbonaceous aerosol production from modern gasoline cars has been observed in tests on US registered vehicles, and in this work for European vehicles. In all of our tests, modern gasoline cars produced more carbonaceous aerosol than modern DPF-equipped diesels. This was true when considering primary particulates only, but the difference is larger when considering the additional SOA production from gasolines, and even larger when considering the effect of low temperatures on emissions. We also show that these measurements are consistent with ambient observations of high fossil SOA fractions in areas with a high fraction of gasoline cars. Our results suggest that as vehicle fleets modernise to include a higher fraction of DPF-diesels the relative contribution from gasoline passenger cars to carbonaceous aerosol will increase. Note that while we draw these conclusions from driving cycles designed to represent real-world driving and from ambient data reflecting an aggregate of all emissions, exceptions are possible. Critically, how much of the carbonaceous aerosol in any region is from one engine technology or the other will depend on the number of vehicles with each type of engine technology in operation and the age of the vehicles (i.e. the number of old, non-DPF vs modern, DPF equipped diesels), which may be region-dependent. Specific cases where diesel passenger cars may emit more carbonaceous aerosol than gasoline may include very long journeys (relative importance of the cold start is lower), operating conditions not investigated here e.g. high speeds (>120 km h^−1^) or extremes of temperature, and outlier vehicles (note the very large ranges in emissions in e.g. Fig. 4). Nevertheless, and since NO_X_ emissions are generally higher from diesel cars, there is a choice between new passenger cars that generally emit less PM and produce less SOA (diesel), or new passenger cars that emit less NO_X_ (gasoline).

## Methods

### Vehicle testing procedure

Experiments were performed at the Vehicle Emissions Laboratory (VELA) of the European Commission Joint Research Centre as described in Platt *et al*.^4^. The climatic test cell (−10 °C to 35 °C temperature range) features two 48″ roller benches (Maha, Germany) and a dilution tunnel (Horiba, Japan) for a constant volume sampler (CVS, average dilution = 40). Gaseous emissions from the vehicles described in SI Tables S3 and S4 were sampled, diluted and collected in Tedlar bags for analysis with an integrated total hydrocarbon analyser (Horiba MEXA 7400 HTRLE) following Directive 70/220/EEC^31^ and its following amendments. Particles were collected from the CVS onto heated filters (Horiba HFU-4770) and measured offline gravimetrically at ambient temperature and directly after the test. Vehicles were driven over the New European Driving Cycle (see SI Fig. 1 and EU Regulation 692/2008 ref. 32) at −7 °C and 22 °C after at least 6 hours soak time at the same temperature of the emission test. Regulated gaseous compounds were measured with the following techniques: total hydrocarbons (THC) with gas chromatography and flame ionization detection GC-FID, carbon monoxide and dioxide (CO, CO_2_) with non-dispersive infrared, nitrogen oxides (NO_X_) with chemiluminescence. In parallel to diluted sampling necessary to fulfil the legislative requirements, we characterised the raw exhaust in real-time (at 1 Hz) during the driving cycles with the same techniques described above. Procedures such as pre- and post-test flushing (30 minutes each) of the transfer lines and dilution tunnel were automatically executed to assure monitored background concentrations below the respective detection limits.

### Smog chamber experimental procedure

Smog chamber experiments are listed in SI Table S5. Prior to all experiments the mobile chamber is cleaned by reducing its volume to ~1 m^3^ and flushing first with ozone (O_3_) and humidified air, illuminating the chamber with UV light for a period of around 1 hour and then flushing with pure dry air for several hours overnight. At the start of each experiment, the bag was filled to approximately two thirds full with humidified air (i.e. leaving a volume free for sample injection during several minutes). Emissions were then sampled directly at the vehicle tailpipe during the NEDC and injected into the chamber using the Dekati ejector dilutor. Dilution provided by the Dekati was ~12, subsequently increased to ~150 by the air inside the smog chamber. The Dekati and sampling lines were heated to 150 °C. Sampling lines are constructed from silica steel. Subsequent to exhaust injection, the chamber was filled to close to maximum volume with pure air. A period of several minutes was allowed for the equilibration inside the chamber and for characterization of the primary emissions. Ozone was added to the chamber to titrate NO emitted by the vehicles to NO_2_. This ensured that conditions were more representative of the troposphere where most NO_X_ is in the form of NO_2_ and increased the reactivity of the gases in the chamber; previous studies have demonstrated that SOA formation only begins following conversion of NO to NO_2_^4, 33^. Note that only enough O_3_ was added to convert the NO, and that an excess was not present before lights-on.

For two experiments (gasoline vehicle 1, Table S5) propene was added prior to lights on in order to adjust the VOC to NO_X_ ratio (with the assumption that propene itself does not form SOA) to enhance OH concentrations, as discussed in Platt *et al*.^4^. For all subsequent experiments nitrous acid (HONO) was added to the chamber via a constant injection stream^34^ to increase OH radical concentrations. Since significant NO_X_ was present in the chamber during all experiments, and since HONO was added, we assume that all experiments were under high NO_X_ conditions. 1 µL (~20 ppbv) of nine-times deuterated butanol (butanol-D9, 98% Aldrich) was injected prior to lights on to quantify OH exposure during the experiments^35^. Butanol-D9 is a unique OH tracer, and measuring its decay, here with the proton transfer reaction time-of-flight mass spectrometer (PTR-ToF-MS), yields time resolved OH concentrations. Following adjustment of the gases, and characterization of the primary emissions, the smog chamber lights were switched on for a period of ~4 hours to initiate photochemistry.

Time-resolved online measurements of particle composition were performed using a high-resolution time-of- flight aerosol mass spectrometer (HR-ToF-AMS, Aerodyne) for organics, nitrate, and ammonium, and an aethalometer (Model AE33, Aerosol d.o.o.) for equivalent black carbon (BC). A detailed description of the working principles of the HR-ToF-AMS and associated data analysis may be found in DeCarlo *et al*.^36^. Data from the HR-ToF-AMS were analysed using high-resolution analysis fitting procedures. Volume data from integrated scanning mobility particle sizer (SMPS, custom built) data from the chamber were transformed into mass concentrations using densities based on the particle chemical composition measured by the HR-ToF-AMS and subtracting BC, to provide a second measurement of the total non-refractory PM mass (i.e. those species quantified in the HR-ToF-AMS). This was used to correct for collection efficiency, CE, which was between 0.5–1.0 throughout all experiments where the non-refractory mass was above detection limit. HR-ToF-AMS data were corrected for background CO_2_ by calibrating the observed CO_2_ in filtered air to external measurements using a cavity ring-down spectrometer (G2401, Picarro).

A correction was applied to the OA mass to account for HR-ToF-AMS measurement interferences of organic aerosol mass in presence of ammonium nitrate (NH_4_NO_3_). As detailed in Pieber *et al*.^37^, nitrate salts interfere with the measurement of particulate CO_2_^+^, by creating an oxidising environment on the particle vaporiser in the HR-ToF-AMS, thereby releasing CO_2_^+^ from carbonaceous residues. We use the proportional relationship *K* between the measured CO_2_^+^ and the NH_4_NO_3_ mass measured during instrument calibrations for data correction. The fractional contribution *K* is linked to the actual NO_3_ mass as a function of time *t* during sampling to determine the absolute contribution of interference based CO_2_^+^ (*K* × measured_NO_3_) to the real organic mass observed as CO_2_^+^ ion:

1 $${\rm{True}}\_{{\rm{CO}}}_{2}^{+}(t)={\rm{measured}}\_{{\rm{CO}}}_{2}^{+}(t)\,-\,K\times {\rm{measured}}\_{{\rm{NO}}}_{3}(t)$$

A *K* value 3–5% was applied based on information gained during instrument calibrations.

Primary aerosol emission was determined from the concentrations in the chamber after the driving cycle was finished and as soon as the CO_2_ signal was stable. Figure S2 shows a comparison of primary mass measured at the chamber (sum of BC and OC) and at the VELA from the CVS. The smog chamber primary aerosol is generally lower than the gravimetric measurements as might be expected since 1) primary emission includes more species than carbonaceous aerosol, e.g. metals, 2) particle losses to the chamber walls during the driving cycles and equilibration period cannot be/are not corrected for, and 3) gravimetric measurements may be affected by adsorption artefacts. Secondary aerosol mass was determined from the increase in OA observed after lights on. The suspended OA concentration C_OA_ is corrected for wall losses (C_OA, WLC_) assuming material lost to the walls does not partition using:

2 $${{C}}_{{\rm{OA}},{\rm{WLC}}}={{C}}_{\mathrm{OA},\mathrm{SUSP}{\rm{.}}}\,(t)+\,{\int }_{0}^{t}k(t)\times {C}_{\mathrm{OA},\mathrm{SUSP}{\rm{.}}}\,(t)\,d(t)$$

Where C_OA,SUSP_ is the OA at time = *t* and *k* an exponential decay constant taken from an exponential fit of BC (determined at λ = 950 nm).

Gas phase composition was measured using a suite of dedicated instruments as also described in Platt *et al*.^4^. Oxygenated VOCs were monitored with the PTR-ToF-MS. The instrument was operated at standard conditions, with a reaction chamber pressure fixed at 2.1 mbar, drift tube voltage and temperature at 500 V and 333 K, respectively, corresponding to an electric field strength applied to the drift tube (E) to buffer gas density (N) ratio of 125 Td. CO_2_, CH_4_ and CO were quantified with the Picarro and total hydrocarbons (THC) were measured with a flame ionization detector (Horiba, THC Monitor APHA-370). The raw THC signal from the smog chamber, [THC]un, is corrected to yield [THC]cor, for response factors, *RF*, of ethanol, acetaldehyde, and formaldehyde^12^ as observed by the PTR-ToF-MS as using:

3 $$\begin{array}{ccc}[THC]cor & = & [THC]un+[C{H}_{3}C{H}_{2}OH]\ast (1-RFC{H}_{3}C{H}_{2}OH)\\  &  & +[C{H}_{3}CHO]\ast \,(1-RFC{H}_{3}CHO)+[HCHO]\ast (1-RFHCHO)\end{array}$$

Several smog chamber experiments were performed where no aging data were obtained (e.g. for G1 at −7 °C), but where data on the primary emission, e.g. from the VELA, were still accessible.

Emission factors (EF) are determined using a carbon mass balance:

4 $$EF=\frac{{\Delta }P}{{\Delta }CO2+{\Delta }CO}\cdot \omega c$$

where *P* is the species of interest, ωc the carbon fraction of the fuel and CO_2_ and CO are in units of carbon mass.

## Electronic supplementary material

Supplementary information
