# Supplementary material for: Gasoline cars produce more carbonaceous particulate matter than modern filter-equipped diesel cars
Source: Sci Rep. 2017 Jul 13;7:4926. doi: 10.1038/s41598-017-03714-9 (PMC5509693; doi:10.1038/s41598-017-03714-9)
Supplement: Supplementary file 1 — Supplementary information [file 41598_2017_3714_MOESM1_ESM.pdf]

Supplementary Information for 'Gasoline cars produce more carbonaceous particulate matter than modern filter-equipped diesel cars'

S. M. Platt[1,2], I. El Haddad[2,*], S. M. Pieber[2], A. A. Zardini[3], R. Suarez-Bertoa[3], M. Clairotte[3], K. R. Dällenbach[2], R.-J. Huang[2], J. G. Slowik[2], S. Hellebust[4,‡], B. Temime-Roussel[4], N. Marchand[4], J. de Gouw[5], J. L. Jimenez[5], P. L. Hayes[6], A.L. Robinson[7], U. Baltensperger[2], C. Astorga[3], and A. S. H. Prévôt[2,*]

[1]Norwegian Institute for Air Research, PO Box 100, 2027 Kjeller, Norway.

[2]Paul Scherrer Institute, Laboratory of Atmospheric Chemistry, CH-5232 Villigen, Switzerland.

[3]European Commission Joint Research Centre, Directorate for Energy, Transport and Climate, Sustainable Transport Unit, 21027 Ispra (VA), Italy.

[4]Aix Marseille Univ, CNRS, LCE, Marseille, France.

[5]NOAA Earth System Research Laboratory, Boulder, CO & CIRES, University of Colorado, Boulder, CO, USA.

[6]Département de Chimie, Université de Montréal, Montréal, Québec, Canada.

[7]Center for Atmospheric Particle Studies, Carnegie Mellon University, Pittsburgh, PA 15213, USA.

*Correspondence to: A. S. H. Prévôt (andre.prevot@psi.ch), I. El Haddad (imad.el-haddad@psi.ch).
‡Now at Central Statistics Office, Cork, Ireland

Modelling secondary organic aerosol yields

The SOA mass parameterisation is based on the absorptive partitioning theory of Pankow[40]:

$$\xi_i = \left(1+\frac{C_i^*}{C_{OA}}\right)^{-1} \qquad \text{Eq. S1}$$

Where ξ_i is the partitioning coefficient of a compound *i*, C_i^* the effective saturation concentration, and C_{OA} is the total concentration of organic aerosol. C_i^* is a semi-empirical property reflecting the vapour pressure of the pure constituents and the way they interact with the organic mixture (effectively including liquid phase activities). Eq. S1 can be parameterised using the volatility basis set (VBS)[14], placing the many semi-volatile organics in a complex mixture into a small number of logarithmically spaced bins of effective saturation concentrations C_i^*, at 298 K, replacing a complex mix of individual compounds with a surrogate *S*, which partitions between gas (*g*) and particle (*p*) :

$$SOA = \sum_{i=1}^{N}[S_i|_{g+p}].\xi_i = \sum_{i}^{N}[S_i|_{g+p}].\left(1+\frac{C_i^*}{C_{OA}}\right)^{-1} \qquad \text{Eq. S2}$$

where *i* represents one bin in *N* total bins. Several generations (*m*) of precursor *j* oxidation via a given oxidant (*Ox*) are possible. The change in concentration in each bin over time (*t*) is the sum of production from generation m-1 and loss from present generation m, of precursor *j*. Both terms depend upon the reaction rate constant with the oxidant, $k_{j,m-1,l}$ and $k_{i,j,m,l}$ for production and loss, respectively. Production of SOA also depends on the yield α. The following equation then describes the evolution of $S_i|_{g+p}$ with time:

$$\frac{d\left[S_{i,j,m}\big|_{g+p}\right]}{dt} = \left\{\sum_l^N \alpha_{i,j,m-1,l}.\,k_{j,m-1,l}.\,[Ox].\left[S_{j,m-1,l}\big|_g\right]\right\} - \left\{k_{i,j,m}.\,[Ox].\left[S_{i,j,m}\big|_g\right]\right\} \quad \text{Eq. S3}$$

l should be regarded as a dummy summation variable, representing the volatility bin of the precursor compound.

Assuming that SOA production proceeds mainly via OH chemistry, with [*Ox*] representing the OH concentration at time *t*, and neglecting the reaction of OH with the particle phase compounds, Eq. S3 can be expressed as a function of the time integrated OH concentration (OH exposure, OH_{EXP} = [*Ox*] × *t* in molec. cm^{-3} h), based on the following first order differential equation:

$$\frac{d\left[S_{i,j,m}\big|_{g+p}\right]}{dOH_{EXP}} = \left\{\sum_l^N \alpha_{i,j,m-1,l}.\,k_{j,m-1,l}.\left[S_{j,m-1,l}\big|_g\right]\right\} - \left\{k_{i,j,m}.\left[S_{i,j,m}\big|_g\right]\right\} \quad \text{Eq. S4}$$

OH_{EXP} is inferred from the decay of butanol-D9 used as a unique OH tracer[35].

Normalising the SOA formation to the initial total carbonaceous emissions (TCE) results in a bulk yield, Y_{bulk}, which may be fitted to the chamber data to give the fitting coefficients, $k_{j,m-1,l}$,$k_{i,j,m}$, and $\alpha_{i,j,m-1,l}$.

TCE contains all non-refractory carbonaceous emissions, including semi-volatile organic compounds (SVOC, $C_i^* = 0.1 - 10^3\ \mu g\ m^{-3}$) , intermediate-volatility organic compounds (IVOC, $C_i^* = 10^4 - 10^6\ \mu g\ m^{-3}$), and volatile organic compounds (SVOC, $C_i^* > 10^6\ \mu g\ m^{-3}$). A major assumption underlying these calculations is that the overall chemical composition of the emissions is relatively consistent between different cars tested. Equations 6/8 were solved using a series of pseudo-Monte-Carlo simulations, relying on the random sampling of the fitting parameters of interest:

(1) The values for number of bins (*N*), oxidation generations (*G*), and number of precursors (*P*) are preselected;

(2) A domain of reasonable inputs is predefined for the mass yield coefficients and reaction rate constants (for a reduction of the computational costs);

(3) Parameters are randomly generated over the domain;

(4) Deterministic computations of Y_{bulk} vs. C_{OA} and OH_{EXP} are performed using the generated inputs;

(5) Parameters representing the chamber data within the noise were retained and presented as probability density functions (Supplementary Fig. 4).

This pseudo Monte Carlo approach was adopted as an alternative to the more classically used least squares regression, as the latter did not capture satisfactorily the measured yields over the entire OH_{EXP} and C_{OA} domains, nor did provide physically meaningful values for $\alpha_{i,j,k,l}$ (often

negative values were obtained). Furthermore, the approach is better suited to a system with many coupled degrees of freedom, offering a direct measure of the uncertainties related to the determination of the volatility distributions and the reaction rate constants. That is, all combinations of parameters that represent the measured yields within our uncertainties are retained, providing probabilistic distributions for these entities. In contrast to least squares analyses, this approach provides several sets of model parameters that represent equally well the observations. Therefore, we used a likelihood ratio test to compare the residuals of different models to the best model with the lowest root mean square error. Several settings were explored for the representation of SOA formation, considering several precursors and several oxidation steps.

Two volatility distributions (Eq. S2) were considered. First, all POA measured by the HR-ToF-AMS was allocated to the lowest volatility bin, implying that this fraction does not partition and hence does not produce any SOA. While this is a crude assumption, recent results suggest that the net amount of primary material lost from the particle phase upon oxidation is similar to the amount that partitioned into the particle phase due the mass increase[41], i.e. increased partitioning of the residual/unreacted gas phase POA compensates lost particle phase POA. In the second setting, we predicted the mass fraction in each bin based on the measured POA mass and using the volatility distribution of May et al. (Supplementary Table 9)[42]. Ultimately, we opted for the simplest model representing our data – i.e. one set of first generation products with one reaction rate constant $k_{j,m-1,l}$ and one volatility set with N=4, [α1, α2, α3, α4] for the saturation concentrations [0.1, 1, 10, 100] – as more complex models did not significantly enhance our ability to represent the measurements. Using this simplest parameterisation, SOA formation is then described by the inverse of the decay in precursor concentration, which depends on the OH/precursor reaction rate, multiplied by the yield term:

$$Y_{bulk} = \sum_i^N \left\{ \left(1 + \frac{C_i^*}{C_{OA}}\right)^{-1} \cdot \alpha_i \right\} \cdot \left(1 - e^{(-k_{OH} \cdot OH_{EXP})}\right) \qquad \text{Eq. S5}$$

The resulting parameters are listed in Supplementary Tables 9/10 for both temperatures, and the model evaluation is represented in Supplementary Fig. 3, for the case of tests at 22°C. The average model errors/experimental reproducibility are estimated to be around 30% and 80% for the tests at 22°C and -7°C, respectively. In Fig. 4A (main text), the yields at both temperatures are presented and compared to those from previous studies. The SOA production rate constant is consistent with apparent precursor reaction rate constants of [1.0-1.4]×10^{-7} and [0.5-1.1]×10^{-7} cm^3 $molec.^{-1}$ h^{-1}, for experiments at 22°C and -7°C, respectively. These reaction rate constants are two to three times greater than expected based on the precursors' structure and reactivity towards OH[43].

Previous experiments on diesel emissions highlight the significance of primary SVOC for SOA production. This is evaluated in Supplementary Fig. 4 in the case of gasoline emissions, by assessing the sensitivity of model results to assumptions regarding POA volatility distribution and the effect of aging thereon. We examined a scenario where POA is entirely non-volatile and another where POA species are semi-volatile and produce SOA upon oxidation. For the second scenario, we considered that POA oxidation either produces secondary vapours with the same volatility distribution as those from the oxidation of VOCs or that oxidation shifts the volatility of POA species by one order of magnitude, according to the scheme suggested in Robinson et al.[44]. For all these scenarios, the results show that similar amounts of SOA are

produced and that the POA initial concentration remains constant with aging, consistent with previous observations reported in the literature[41]. Such results indicate that OA evolution with aging is not significantly sensitive to the assumed POA volatility distribution and suggest that primary SVOCs contribute minimally to SOA production, even when assumed to be very efficient SOA precursors (i.e. the Robinson et al. scheme[44] results in SVOC SOA yields of 68%).

This SOA model described here is very similar to the VBS model developed by Carnegie Mellon University for modelling emission aging (e.g. Ref.[38] and references therein); it permits tracking the oxidation products of different precursors. However, a major advance compared to previous models is that it does not require *a priori* knowledge of the precursors' structure and reaction rate constants; instead, the volatility distribution of surrogate compounds formed from one or several precursors at different reaction generation is fitted to represent best the measurements. Such an approach is suited for a complex mixture of unknown SOA precursors, such as gasoline exhaust emissions.

Loss of vapours to the chamber walls

Recent laboratory experiments suggest that SOA formation may be substantially suppressed due to losses of SOA-forming vapours to the clean Teflon chamber walls[45-47]. An indication that these processes might be of importance in our case is that SOA production is significantly faster than expected based on VOC decay rates, e.g. for compounds with carbon number below C_{15} and a low O:C ratio, estimated k_{OH} ranges between [4-8]$\times 10^{-8}$ cm^3 $molec.^{-1}$ h^{-1} [Ref. 43]. While a less likely scenario where the emitted fumes are highly reactive cannot be excluded, all the more since the structure of these precursors is unknown, we investigate the effect of the loss of vapours to the chamber walls based on the assumption that SOA production rates should be proportional to the precursors' expected k_{OH}. We note that the thorough quantification of these effects is beyond the scope of this study and yields will not be corrected for vapour wall losses in order to be compared with previously reported yields (Fig. 4, main text), but we will explore in the following how such effects can alter estimated reaction rates and yields.

Calculations are performed to determine the magnitude of the wall-loss bias, F_{wall}, a scaling factor by which the yields determined here have to be multiplied to obtain the true yields. Vapours can be lost to the walls either by adsorption, where walls act as a vapour sink or by absorptive partitioning[48], where vapours and material condensed on the walls exist at equilibrium following Henry's law. While the two processes are significantly different, under our conditions they induce similar effects on the equilibrium gas-phase vapour concentrations and consequently on the net change in SOA. That is, Matsunaga and Ziemann[48] estimated an equivalent absorptive organic mass concentration of the walls of ~10 mg m^{-3} (C_W=2-24 mg m^{-3}), up to three orders of magnitude higher than the particle loadings measured in our chamber (10-50 µg m^{-3}). Consequently, even when considering an absorptive partitioning scenario most of the SOA forming vapours (C^*=1-100 µg m^{-3}) will eventually reside on the walls. Therefore, we can simulate the wall losses of secondary vapours for both scenarios as a first-order process, characterized by the first-order wall-loss coefficient k_w and an average lifetime for vapour wall loss τ_w (τ_w can also be regarded as an equilibration time scale:

$$\tau_w=1/(k_w+k_g)=1/(k_w+k_w\times C^*/C_W)=1/k_w). \qquad \text{Eq. S6}$$

where k_w and τ_w are mainly dependent on the wall accommodation coefficient, α_w, unknown for our chamber. In this calculation, we neglect the wall losses of primary precursors as we suggest that these are mainly VOCs ($C^*=10^7$ µg m^{-3}>>C_W, Table S9/10). We also consider gas-particle partitioning to be instantaneous, as with our particle condensational sink (CS) gas-to-particle equilibration time scales (τ_{gp}) are expected to be significantly shorter than τ_w reported to be on the orders of 10-100 min [20]:

$$\tau_{gp} = \frac{1}{\text{CS}} = 1/\left(2\pi N_p d_p D_{gas} F\right) \qquad \text{Eq. S7}$$

In this expression, $D_{gas} = 10^{-5}$ m^2 s^{-1} is the gas-phase molecular diffusivity, d_p=0.25 µm is the particle diameter, N_p= 5×10^3 cm^{-3} is the particle number concentration. The Fuchs-Sutugin factor F

$$F = (1+Kn)/(1+0.3773Kn+1.33Kn.(1+Kn)/\alpha_p) \qquad \text{Eq.S8}$$

is a correction factor for non-continuum effects, with Kn being the Knudsen number ($Kn=2\lambda/d_p$, where λ is the gas mean free path = 0.15 µm) and α_p is the particle mass accommodation coefficient. Assuming a reasonable value of α_p = 0.1[Ref. 49] results in equilibration time scales of 2.5 min < expected τ_w and under such conditions τ_{gp} and τ_w would only become competitive, when α_p < 0.01. This simplification would lead to an underestimation of the gas losses, especially for the case of the least volatile fraction and thus losses presented here should be regarded as lower estimates.

This leaves three unknowns: k_w, F_{wall} and the 'true' k_{OH}. Previous studies seeking the quantitative assessment of vapour wall losses have used a single precursor with a known reaction rate, while here emissions contain a complex mix of unknown precursors. Therefore, the precursor reaction rate constants have to be estimated based on their structure. For compounds with carbon number below C15 and a low O:C ratio, estimated k_{OH} ranges between [4-8]×10^{-8} cm^3 molec.$^{-1}$ h^{-1} [Ref. 45]. Accordingly, these k_{OH} values were fixed in the model and k_w/τ_w and F_{wall} were then optimised to fit the observed SOA mass and production rates. The optimal values for τ_w are 30 minutes and 90 minutes, with a corresponding bias of factor 2.7 and 1.5, for k_{OH} = 4 and 8×10^{-8} cm^3 molec.$^{-1}$ h^{-1}, respectively. These values are in excellent accordance with those reported in Zhang et al.[45].

For OH_{EXP} less than 25×10^6 molec. cm^{-3} h, i.e. 1 day of aging at OH concentrations of 10^6 molec. cm^{-3}, the uncorrected model may underestimate gasoline SOA contributions by up to 50% in Fig. 4A, irrespective of the assumed parameters, using

$$\text{SOA}_{\text{true}}/\text{SOA} = \text{F}_{\text{wall}} \times (1-\exp(-k_{\text{OH, true}} \times \text{OH}_{\text{EXP}}))/(1-\exp(-k_{\text{OH, fitted}} \times \text{OH}_{\text{EXP}})) \qquad \text{Eq. S9}$$

Conversely, for aged air masses, the model bias is highly dependent on these parameters and the uncorrected model may lead to more than a factor 2 underestimation. This correction is not applied to the data presented in this study as it represents a preliminary effort to assess this effect, and to allow for better comparison with other studies. However, it does serve to highlight one reason for the relatively low estimated contributions from fossil sources, both gasoline (Fig. 4A) and diesel (Supplementary Fig. 7).

Comparison between yields at 22°C and at -7°C

In addition to determining the volatility distributions of SOA species formed at 22°C and at -7°C, the examination of the yields' temperature dependence is indispensable for a better representation of SOA production from vehicle emissions under winter conditions. The temperature dependence of C^* of different bins can be specified assuming vapour pressures vary according to the Clausius-Clapeyron equation, dependent on the compounds' enthalpies of evaporation, ΔH_{vap} (J mol^{-1}) such that

$$C^*(T) = C^*(T_{ref}).\left(\frac{T_{ref}}{T}\right).\exp\left[-\frac{\Delta H_{\mathrm{vap}}}{R}.\left(\frac{1}{T}-\frac{1}{T_{ref}}\right)\right] \quad \text{Eq. S10}$$

In this expression, T_{ref} and T are the reference and actual temperatures (K) at which the experiments are performed and R is the ideal gas constant (J mol^{-1} K^{-1}). Indeed, this analysis is based on the underlying assumption that the gas phase composition of the emissions is similar at both temperatures. In practice, we determined ΔH_{vap} by fitting both the increase and decrease of yields with the decrease and increase in temperature, respectively; i.e. considering T_{ref} = 22°C ; T = -7°C and T_{ref} = -7°C ; T = 22°C. For the first case, the volatility distribution function at volatility bins higher than determined here (i.e. $C^* > 100$ µg m^{-3}) are required for the fitting. The extrapolation to higher volatility bins was achieved by assuming that the volatility distributions follow a Gaussian function, with a total yield constrained to 1.4 (this takes into account the addition of oxygen to the organic compounds with oxidation). Results show that the temperature dependence of SOA yields is consistent with physically unrealistically low values for ΔH_{vap}=19 KJ mol^{-1}, corresponding to an average increase in yields by 2±0.4% K^{-1} with the temperature decrease, in the temperature range [-20–40]°C. Similarly low ΔH_{vap} values have often been previously obtained from chamber studies of single anthropogenic and biogenic precursors and subsequently utilised to describe the overall sensitivity of OA to changes in temperature [50-52]. It is worth noting that the use of such low values is only valid as a simplification when attempting to represent the bulk aerosol, as few lumped components (such as is the case here and in chemical transport models). However, as was pointed out by Donahue et al.[14], when individual compounds (or a wider range of volatility bins) are used, the utilization of larger ΔH_{vap} values, consistent with the properties of individual compounds, is more appropriate.

Extrapolation of chamber results to ambient

We focused on the investigation of the sources of anthropogenic SOA from fossil emissions (f_{SOA}) measured during CAlNex 2010, in Pasadena, CA. Based on the ^{14}C analysis presented in Zotter et al.[27], 70 % of the SOA enhancement (increase in concentration over the course of the day) may be attributable to anthropogenic fossil-fuel activity. Since the concentration of SOA precursors from vehicle emissions is not accessible, we utilized a number of tracers together with yields determined in our chamber and in previous studies to estimate the contribution of passenger car exhaust to ambient f_{SOA}. These estimates are presented in Fig. S5 and discussed in the main manuscript.

Toluene and HOA (hydrocarbon-like OA, a surrogate of vehicle emissions retrieved by factor analysis) can be used as tracers to estimate the contribution of gasoline emissions from our chamber results. Bahreini et al.[23] estimated that 30% of HOA in the ambient L.A. atmosphere

is from gasoline cars, the remainder being from diesel. By equating HOA with primary organic aerosol (POA), an estimate of the gasoline car f_{SOA} is given by the ratio SOA/POA vs. OH exposure in the chamber × ambient HOA vs. ambient OH exposure. Toluene accounts for ~10% of gasoline exhaust THC, total ambient gasoline THC may thus be estimated as ~10 × ambient toluene. Here we assume emissions of toluene are predominantly from gasoline vehicles, on the basis that 1) Borbon et al.[28] demonstrate that for L.A (and also Paris, France) toluene strongly correlates with ambient CO, emissions of which are dominated by vehicles and 2) our PTR-ToF-MS measurements show limited emission of toluene from the diesel cars (Fig. 2). Gasoline SOA is then estimated from the bulk yield as a function of OH exposure (Eq. S5). An estimate was also done using yields for LEV1 and LEV2 gasoline passenger cars and reaction rate constants for THCs from LEV1 and LEV2 gasoline cars in Gordon et al.[3].

It is critical to compare the above estimates to those using SOA yields from previous studies, in order to assess the contribution from other fossil activities to SOA. While results from Chirico et al.[15], Gordon et al.[3] and this work underscore the effectiveness of catalyst coated DPFs at reducing SOA formation from diesel vehicles, we expect that a part of SOA is related to non-DPF-equipped diesel vehicles. To estimate the contribution of these vehicles, we use HOA as a tracer (70% from diesel vehicles), in the same way as for gasoline cars and yields in Gordon et al.[3]. Similar to Chirico et al.[15], this study reports SOA/POA ratios ranging between 0.4 and 1.3 (geometric mean ± geometric standard deviation), at an OH_{EXP} of 15×10^{6} molec. cm^{-3} h. Assuming that the reaction rates of diesel exhaust ranges between $[4\text{-}8]\times10^{-8}$ cm^{3} $molec.^{-1}$ h^{-1}, we estimate that their contribution to total fossil SOA ranges between 4 and 15%, significantly lower than the gasoline emission contribution to SOA production.

In addition to the uncertainties associated with SOA yields, there are several uncertainties in each of these models, which may explain the lack of closure of gasoline and diesel exhaust with total f_{SOA}. For example, a significant contribution of evaporative emissions to ambient toluene cannot be neglected and would affect our estimations depending on whether committed compounds from evaporative sources have higher or lower yields than gasoline car emissions. For example, ambient toluene from evaporative emissions of gasoline would lower the estimated contribution (since the yield is lower, as shown in Fig. 3A). Moreover, SOA/POA ratios may vary considerably between vehicles. There is also a large uncertainty associated with the ambient OH exposure. Finally, while closure with the ambient data is not achieved, the important information is the split between f_{SOA} from gasoline and diesel vehicles showing that gasoline is the larger fraction, rather than the total.

Fractional contribution of diesel vehicles to passenger car particulate matter

Equation 4 (main text, methods) can be rearranged to give the total emitted pollutants, where the emission factor is known (since CO emissions are negligible compared to CO_2 emissions). Using the present fleet average emission factors[8] and an average emission factor allowing for a given fraction having emissions factors of the Euro 5 passenger cars from this work ($EF_{Diesel,Av}$, $EF_{Gasoline,Av}$), the fractional contribution of diesel vehicles to PM ($Diesel_{PM\_Share}$) as a function of diesel vehicle fraction (f_{Diesel}) and fraction of modern vehicles can be determined:

$$Diesel_{PM\_Share} = \frac{f_{Diesel} \cdot EF_{Diesel,Av}}{f_{Diesel} \cdot EF_{Diesel,Av} + (1 - f_{Diesel}) \cdot EF_{Gasoline,Av}} \quad \text{Eq. S11}$$

A projection for the European fleet between 2005 and 2030 is possible using modelled CO_2 emissions as a function of vehicle emission standard[30], by equating the ratio CO_2 emissions to the ratio of fuel consumption, given similar fuel carbon content and that CO_2 is by far the largest combustion product (>95%) . Since emissions for non-DPF diesel are much higher than those from DPF-equipped diesel, the diesel PM share is dominant above only a small fraction of non-DPF-equipped diesels (where the second term in the denominator becomes insignificant) and errors in the emission factors from gasoline passenger cars and DPF-equipped diesels do not have a large impact. Electric vehicles, expected to increase as a fraction of the fleet have an emission factor EF=0, as do hybrid vehicles while consuming electric power. They would therefore not alter the relative contribution of diesel to exhaust PM.

Supplementary Figures

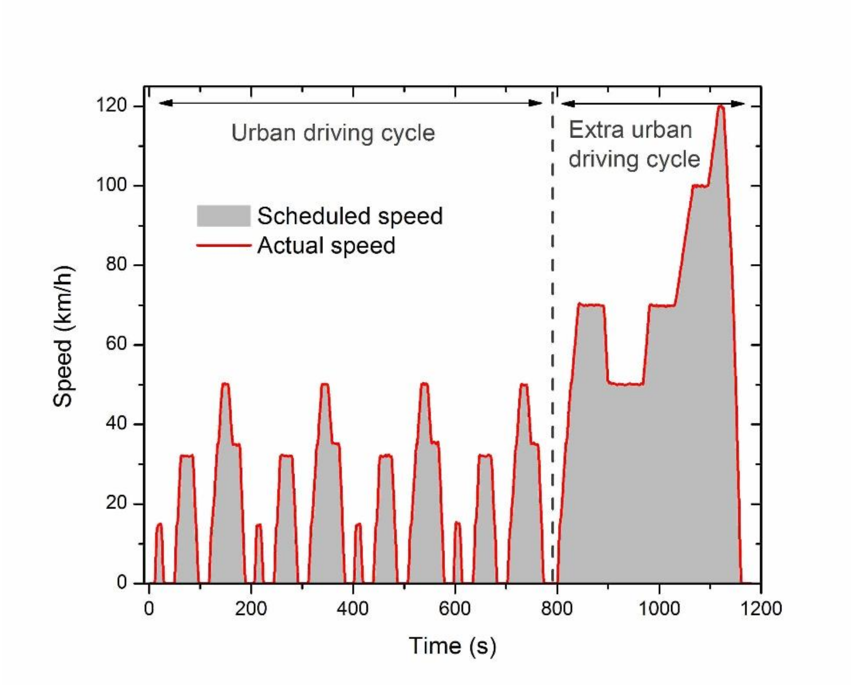

Supplementary Figure 1. The New European Driving Cycle, showing target speed as a function of time and the division between urban and extra urban phases.

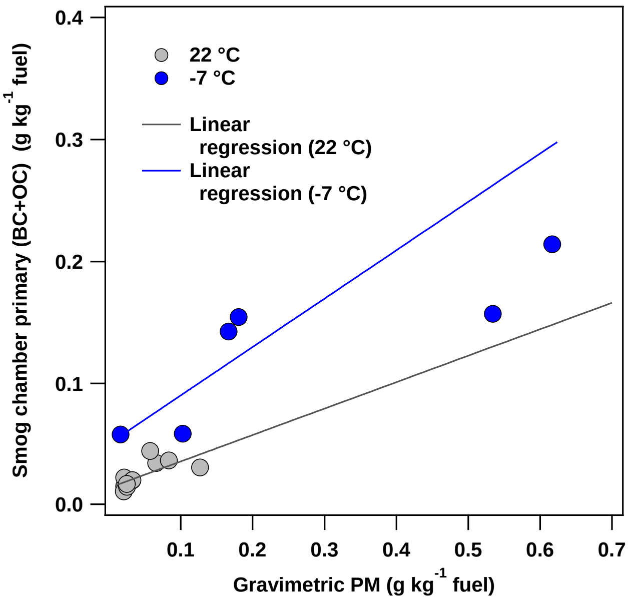

Supplementary Figure 2. Comparison of gravimetric (from the constant volume sampler, CVS) and online smog chamber (sum of black carbon, BC, and organic carbon OC) measurements of primary PM from the gasoline vehicles of this study (G1, G2, G3, Supplementary Table 2). Orthogonal distance linear regressions are shown at each temperature. At 22ºC: smog chamber PM= 0.01+0.23 × gravimetric PM (r^2=0.5), At -7ºC: smog chamber PM= 0.05+0.40 × gravimetric PM (r^2=0.6).

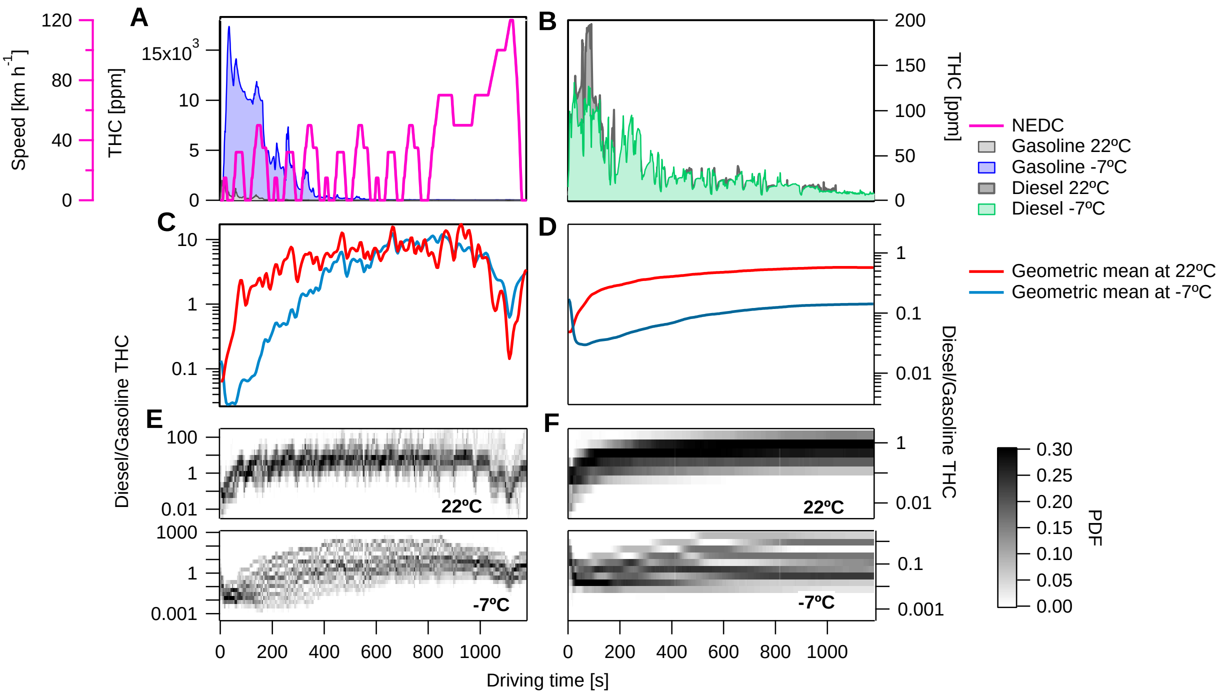

Supplementary Figure 3. Time resolved total hydrocarbon emissions from Euro 5 gasoline and diesel vehicles. A, B, Time dependent comparison of THC emissions from Euro 5 gasoline and diesel passenger cars during the new European driving cycle (NEDC, pink line), respectively, at 22 and -7°C **C,** Geometric mean of the ratio diesel/ gasoline THC emissions at 22 and -7°C (red and blue, respectively) during the NEDC. **D,** Ratio of the cumulative emissions over the full NEDC. Geometric means are calculated from the ratio of two probability distributions. Lower panels show the diesel/ gasoline ratio as a probability density functions **E,** time resolved, and **F,** integrated.

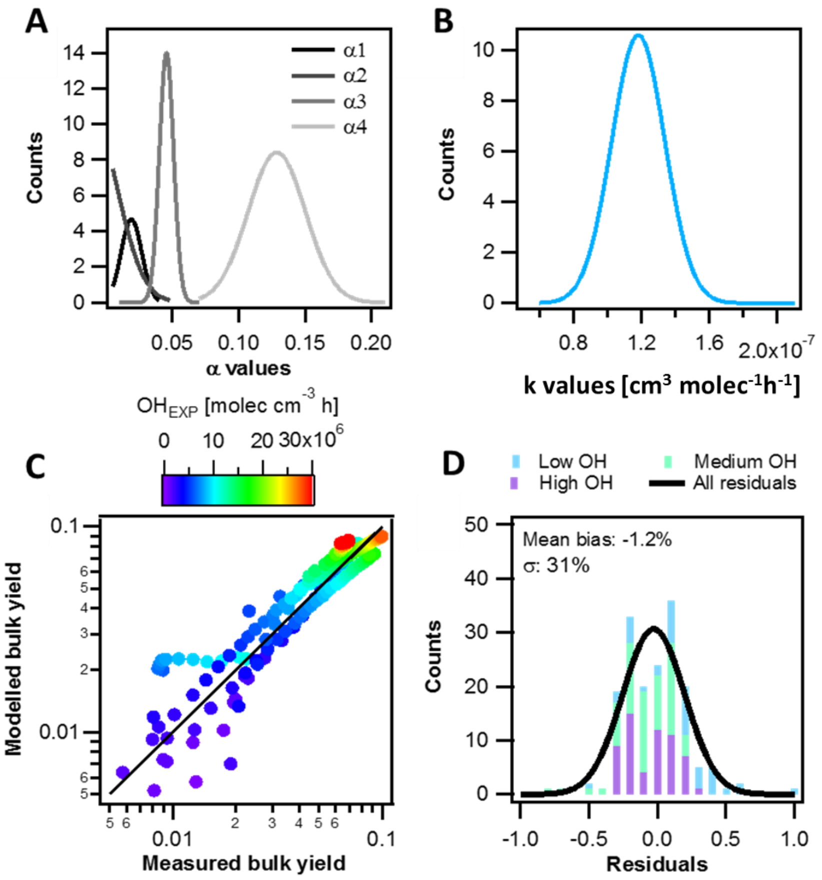

Supplementary Figure 4. Model output and evaluation for experiments at 22°C. Panels **A and B** represent the probability density functions for 50 sets of model parameters, the volatility set (α values) and the reaction rate constant k_{OH}, respectively. Panel **C** represents a scatter plot between measured and modelled bulk yields (SOA formed to total initial hydrocarbons) obtained from one of the models [α1=0.0023; α2=0.045; α3=0.042; α4=0.1; k_{OH} =1.14×10^{-7} cm^3 molec.$^{-1}$ h^{-1}], for 6 experiments including 3 cars. In Panel **D** we evaluate the model residuals for the same model, showing that the model reproduces accurately the data (accuracy~-1.2%; precision~31%), for low (<5×10^6 molec. cm^{-3} h), medium (>5×10^6 molec. cm^{-3} h; <15×10^6 molec. cm^{-3} h) and high (>15×10^6 molec. cm^{-3} h) OH exposures.

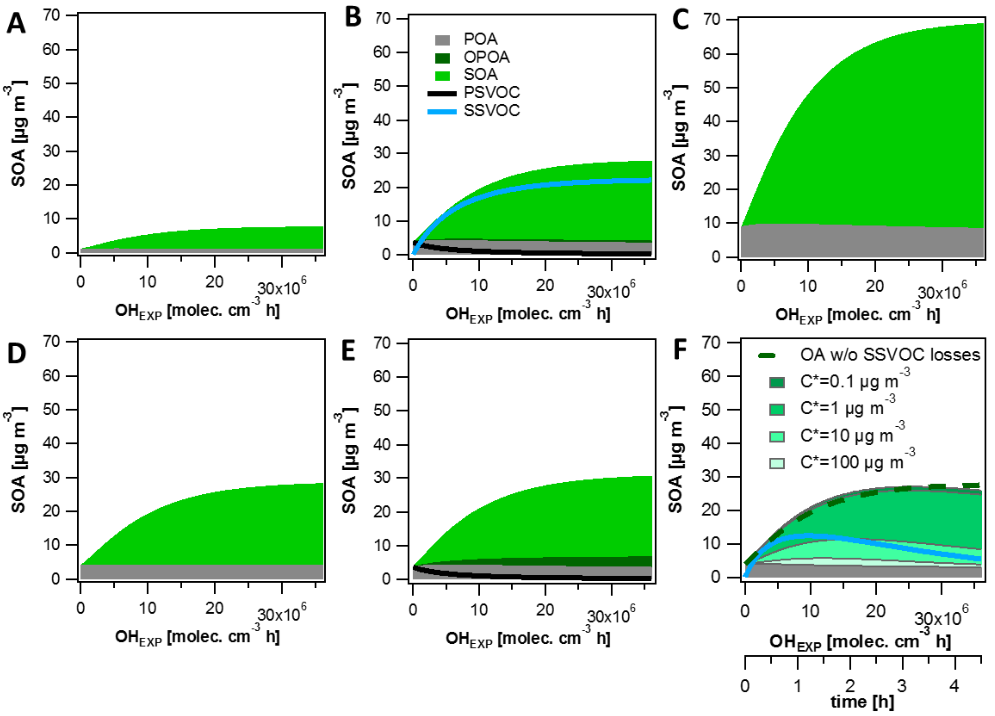

Supplementary Figure 5. Modelled SOA for experiments at 22°C considering different scenarios. PSVOC and SSVOC denote primary and secondary semi-volatile organic vapours, while OPOA is the part of SOA produced from the oxidation of primary SVOC and IVOC compounds. In panels **A, B, C, E** and **F**, the volatility distribution of primary SVOC and IVOC from May et al.[53] was used, while in panel **D**, POA was considered non-volatile under our conditions. In panels **A**, **B** and **C**, the simulations were run at initial TCE concentrations of 100, 250 and 500 µg m^{-3}, respectively. For the other panels a TCE of 250 µg m^{-3} was used. In panel **E**, we considered that the reaction of primary SVOC and IVOC vapours shifts their volatility by one order of magnitude, according to the scheme suggested in Robinson et al.[44]. In panel **F**, we considered the loss of secondary vapours to the walls. We distinguish by different green tonalities SOA surrogates of different volatility bins in order to illustrate the decrease in the contribution of the most volatile compounds upon the loss of their gases on the walls. In this scenario, we could faithfully reproduce the observation (dark green line), by assuming more realistic reaction rate constants (here $k_{OH} = 6\times10^{-8}$ cm^3 molec.$^{-1}$ h^{-1}), if the yields were to be 1.9 times higher with average lifetimes of secondary vapours of one hour. The May et al.[53] is based on quartz filter measurements therefore only captures a small fraction of the IVOC emissions[49].

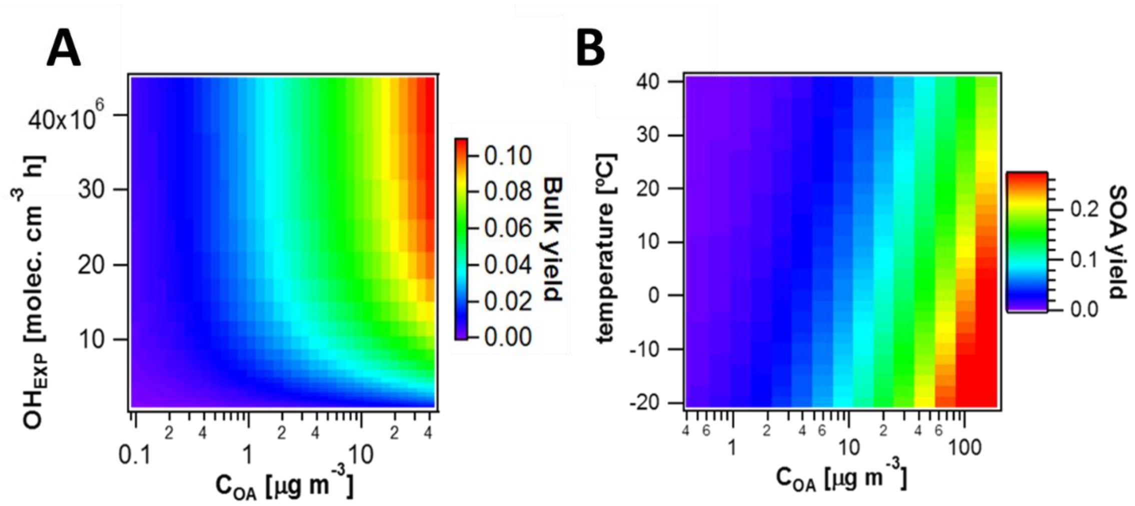

Supplementary Figure 6: Secondary organic aerosol yields from Euro 5 gasoline car emissions. A, Secondary organic aerosol (SOA) yields (colour scale) as a function of suspended organic aerosol (C_{OA}) and OH. **B,** SOA yields (colour scale) as a function of C_{OA} and temperature. The C_{OA} and OH exposure ranges are those spanned during the experiments**.**

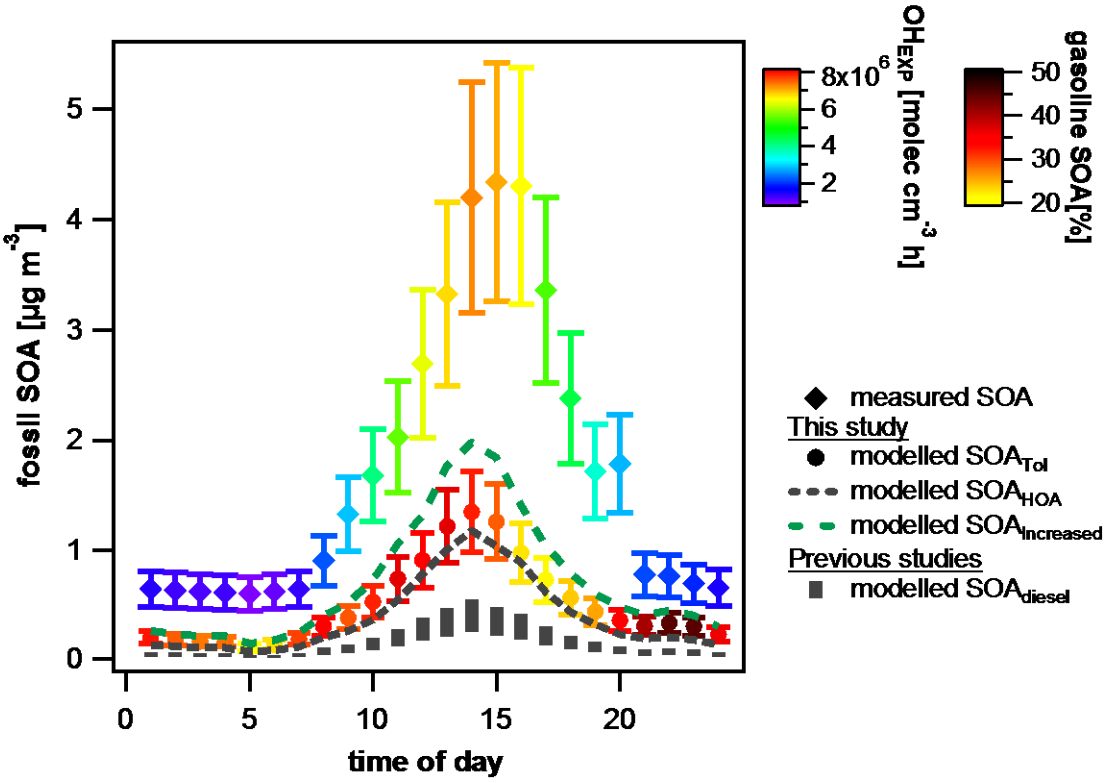

Supplementary Figure 7. Measured ambient fossil SOA as a function of OH exposure (red/blue colour scale) and time of day. Fossil SOA from gasoline vehicles is determined using yields determined in this study and determined from ambient L.A. toluene concentration reported (SOA_{TOL}), coloured by fractional contribution to the total (yellow/ brown colour scale). Also shown: estimated fossil SOA from gasoline vehicles using the SOA/POA ratio (SOA_{HOA}) observed in this study and the yield expected from the ambient aromatic hydrocarbon concentrations (SOA_{AR}). The influence of vapour losses is given by the upper dashed line. Also shown, estimates from previous studies of the diesel contribution to fossil SOA based on SOA/POA[50] and vehicular SOA[3].

Supplementary Tables

Supplementary Table 1. Euro 5 and Californian LEV2 emission standards for passenger cars [g km^{-1}]. LEV2 standards are converted from g mile^{-1}, limits for vehicles with less than 50000 miles/ 5 years old are shown. Formaldehyde (limited for LEV2) is omitted.

	Date	CO	THC	NMHC	NO_X	HC+ NO_X	PM
**Euro 5**							
Diesel	09/2009	0.50	-	-	0.180	0.230	0.005
Gasoline	09/2009	1.0	0.10	0.068	0.060	-	0.005*
**LEV2 (g km^{-1})**							
All light duty	11/1998	2.1	-	0.047	0.031	-	-

*Applies to direct injection vehicles only

Supplementary Table 2: Overview of instrumentation used at the smog chamber. Adapted from Platt et al., 2013[Ref 4]

Parameter	Instrument	Manufacturer and model
Non-refractory particulate matter (Organics, nitrate, sulfate, ammonium, chloride)	High resolution time-of-flight aerosol mass spectrometer (HR-ToF-AMS)	Aerodyne
Particle number and size distribution	Scanning mobiliy particle sizer (SMPS), long DMA+ condensation particle counter CPC	Home built SMPS with TSI 3022 CPC
Black carbon	Aethalometer	Aerosol AE33
NO+NO_Y	NO_X monitor	Thermo environmental 42C
NO+NO_Y	NO_X monitor	Monitor labs 9841A
O_3	UV photometric analyser	Monitor labs 49C
CO_2/ CO/ CH_4/ H_2O	Cavity ring down spectrometer	Picarro G2401
Total hydrocarbon (THC)	THC monitor (flame ionisation detector FID)	Horiba APHA-370
Volatile organic compounds (VOC)	Proton transfer reaction time-of-flight mass spectrometer	Ionicon analytic
Relative humidity	Dew point hygroscope	Rotronic SC 05
Temperature	Thermocouple	Messelemente Type K

Supplementary Table 3. Technical details of the gasoline vehicles used in this study. All vehicles were equipped with three-way catalysts. TWC=three-way catalyst, DI=direct injection, PFI=port fuel injection.

Designation	G1	G2	G3	G4	G5	G6	G7	G8	G9	G10	G11
After-treatment	TWC	TWC	TWC	TWC	TWC	TWC	TWC	TWC	TWC	TWC	TWC
Fuel Injection	PFI	DI	DI	DI	DI	PFI	PFI	PFI	PFI	PFI	DI
Displacement [cm^3]	875	1390	1997	1984	1995	1242	1595	1242	1490	1368	1984
Power [kW]	62.5	90	135	132	105	44	75	51	82	57	132
Odometer [km]	1376	38951	6738	1441	8353	1909	7285	13699	35	42146	12461

Supplementary Table 4. Technical details of the diesel vehicles used in this study. DOC=diesel oxidation catalyst, DPF= diesel particle filter.

Designation	D1	D2	D3	D4	D5	D6
After-treatment	DOC + DPF	DOC + DPF	DOC + DPF	DOC + DPF	DOC + DPF	DOC + DPF
Displacement [cm^3]	1968	2987	1598	1995	1598	1248
Power [kW]	103	140	77	130	55	55
Odometer [km]	27410	2848	6320	4667	1058	6402

Supplementary Table 5: List of smog chamber experiments by date and vehicle tested, temperature, VOC/NO_X ratio, average OH radical concentration, and organic carbon (OC) loadings immediately prior to lights on.

**Date**	**Vehicle**	**Temperature °C**	**VOC/NO_X**	**OH 10^6 cm^{-3}**	**Loading µg m^{-3}**
13.10.2011	G1	22	6.1	1.2	20.42
14.10.2011	G1	22	5.6	2.1	5.92

12.02.2013	G2	22	4.1	-	1.01
13.02.2013	G2	22	3.1	2.9	0.16
14.02.2013	G2	22	3.2	9.1	2.08
15.02.2013	G2	22	4.5	7.5	3.04
18.02.2013	G2	-7	3.8	1.5	36.48
19.02.2013	G2	-7	15.7	1.1	0.29
20.02.2013	G2	-7	10.9	5.3	5.34
21.02.2013	D1	22	0.9	5.3	-
22.02.2013	D1	22	1.0	11.1	-
25.02.2013	D1	22	0.9	8.1	-
26.02.2013	D1	-7	0.2	1.0	-
27.02.2013	D1	-7	0.2	2.1	-
28.02.2013	D1	22	1.1	9.1	-
01.03.2013	D2	22	0.2	6.4	-
04.03.2013	D2	22	0.1	-	-
05.03.2013	D2	22	0.3	10.0	-
06.03.2013	D2	22	0.3	16.7	-
07.03.2013	D2	-7	0.0	20.0	-
08.03.2013	D2	-7	0.5	17.6	-
11.03.2013	G3	22	2.7	37.5	4.10
12.03.2013	G3	22	3.1	7.9	7.60
13.03.2013	G3	-7	3.3	11.4	31.10
14.03.2013	G3	-7	3.3	14.0	2.00

Supplementary Table 6. Average emission factors (EF, g kg^{-1} fuel) of organic carbon (OC) black carbon (BC), particulate matter (PM), and total hydrocarbon (THC) at 22ºC reported in this study, and number of repeats (*n*) performed in the determination of each EF. EFs in g km^{-1} are given in parenthesis.

**Vehicle**	**EF ×10^3**				***n***
	**OC**	**BC**	**PM**	**THC**	
This work					
G1	7.8 (0.4)	9.5 (0.5)	26.8 (1.4)	1068 (55.6)	2
G2	2.4 (0.2)	15.1 (1.1)	24.9 (1.8)	435 (31.7)	4
G3	7.2 (0.5)	29.1 (2.0)	83.3 (5.73)	334 (23.0)	3

D1	2.0 (0.1)*	1.0 (0.0)*	3.0 (0.14)	952 (43.9)	4
D2	1.1 (0.1)*	0.1 (0.0)*	1.4 (0.09)	250 (15.6)	4
D3			22.1 (0.17)	6.6 (51)	4
Clairotte et al.[9], converted from mg km^{-1}					
G4	-	-	3.1	210	3
Dardiotis et al.[51], converted from mg km^{-1}					
G5	-	-	-	223	1
G6	-	-	-	420	5
G7	-	-	-	179	2
G8	-	-	-	221	2
G9	-	-	-	196	2
G10	-	-	-	213	5
G11	-	-	-	376	3
D4	-	-	-	243	4
D5	-	-	-	372	2
D6	-	-	-	0.20	3

*Below detection on all tests, average of test specific detection limits given

Supplementary Table 7: Average emission factors (EF, g kg^{-1} fuel) of organic carbon (OC) black carbon (BC), particulate matter (PM), and total hydrocarbon (THC) at -7°C reported in this study, and number of repeats (*n*) performed in the determination of each EF. EFs in mg km^{-1} are given in parenthesis.

**Vehicle**	**EF ×10^3**				***n***
	**OC**	**BC**	**PM**	**THC**	
This work					
G1	124.5 (4.2)	24.0 (0.8)	173 (5.8)	2520 (84.54)	2
G2	23.8 (0.6)	252.4 (6.1)	592 (14.22)	8954 (215.2)	3
G3	2.3 (0.6)	56.2 (15.83)	59 (16.62)	1252 (352.7)	3
D1	1.2[†] (0.02)	0.1* (0.0)	0.8 (0.26)	651 (12.4)	2
D2	4.3* (0.1)	0.1* (0.0)	4.9 (0.72)	324 (4.4)	2
Clairotte et al.[9], converted from mg km^{-1}					
G4	-	-	0.0031	1.49	3
Dardiotis et al.[51], converted from mg km^{-1}					
G5	-	-	-	444	3
G6	-	-	-	1164	2

G7	-	-	-	1000	2
G8	-	-	-	731	1
G9	-	-	-	1199	2
G10	-	-	-	1196	2
G11	-	-	-	935	2
D4	-	-	-	3007	2
D5	-	-	-	978	2
D6	-	-	-	1632	3

*Below detection on all tests, average of test specific detection limits given

†Only one value from one test not below detection; *n*=1

Supplementary Table 8: SOA formation from gasoline vehicles (mg carbon (C) kg^{-1} fuel) at 22 and -7 ºC at OH exposure=10^{7} cm^{-3}h, and number of repeats (*n*). SOA formation in mg km^{-1} is given in parenthesis.

Vehicle	SOA formation	*n*
22ºC		
G1	44.5 (2.33)	2
G2	5.7 (0.42)	2
G3	28.0 (1.92)	3
-7 ºC		
G1	-	-
G2	27.0 (0.65)	2
G3	71.22 (20.07)	3

Supplementary Table 9. Estimated volatility distribution of primary gasoline emissions, using the measured concentrations of POA and THC and the volatility distribution of POA from May et al. [40].

$Log_{10}(C_i^*)$	C_i^*	$\alpha_{i,1,0}$
-2	0.01	5.0×10^{-3}
-1	0.1	4.3×10^{-3}
0	1	5.0×10^{-3}
1	10	8.7×10^{-3}
2	100	5.0×10^{-3}
3	1000	1.0×10^{-3}
4	10000	6.7×10^{-4}
5	100000	3.3×10^{-4}

6	1000000	3.6×10^{-3}
7	10000000	9.65×10^{-1}

Supplementary Table 10. Parameterised yield fractions and reaction rate constant (in cm^3 molec.$^{-1}$ h^{-1}) for experiments at 22 and -7°C.

Parameter	22°C		-7°C	
	range	Recommended	range	Recommended
α1	0.005-0.02	0.0023	0.001-0.005	0.0040
α2	0.005-0.05	0.045	0.001-0.007	0.0044
α3	0.04-0.05	0.042	0.02-0.1	0.026
α4	0.1-0.15	0.1	0.34-0.66	0.47
k_{OH}	$[1.0-1.4]\times10^{-7}$	1.14×10^{-7}	$[0.5-1.1]\times10^{-7}$	0.53×10^{-7}

Supplementary Table 11. Ambient carbonaceous aerosol (black carbon, BC, hydrocarbon-like organic aerosol, HOA, and secondary organic aerosol, SOA) concentrations and errors. Source reference for data is given

Location	Carbonaceous aerosol [μg m^{-3}]			Error [μg m^{-3}]			Reference
	BC	HOA	SOA	BC	HOA	SOA	
Marseilles (France)	1.8	0.8	0.7	0.4	0.1	0.1	El-Haddad et al.[54,55]
Barcelona (Spain)	1.4	0.9	1.0				Minguillon et al.[56]
Paris (France)	1.1	0.9	0.6	0.3	0.1	0.2	Crippa et al.[57]
Zurich (Switzerland)	1.3	0.5	0.6	0.3	0.2	0.4	Zotter et al.[58]
Bakersfield (US)	0.3	0.6	2.5				Liu et al.[59]
Pasadena (US)	0.6	1.4	2.9	0.2	0.2	0.5	Zotter et al.[27]

References

40 Pankow, J. F. An absorption model of gas/particle partitioning of organic compounds in the atmosphere. *Atmospheric Environment* **28**, 185-188 (1994).

41 Presto, A., Gordon, T. & Robinson, A. Primary to secondary organic aerosol: evolution of organic emissions from mobile combustion sources. *Atmospheric Chemistry and Physics* **14**, 5015-5036 (2014).

42 May, A. A. *et al.* Gas-particle partitioning of primary organic aerosol emissions:(1) Gasoline vehicle exhaust. *Atmospheric Environment* **77**, 128-139 (2013).

43 Kwok, E. S. & Atkinson, R. Estimation of hydroxyl radical reaction rate constants for gas-phase organic compounds using a structure-reactivity relationship: an update. *Atmospheric Environment* **29**, 1685-1695 (1995).

44 Robinson, A. L. *et al.* Rethinking organic aerosols: Semivolatile emissions and photochemical aging. *Science* **315**, 1259 (2007).

45 Zhang, X. *et al.* Influence of vapor wall loss in laboratory chambers on yields of secondary organic aerosol. *Proceedings of the National Academy of Sciences* **111**, 5802-5807 (2014).

46 Ye, P. *et al.* Vapor wall loss of semi-volatile organic compounds in a Teflon chamber. *Aerosol Science and Technology* **50**, 822-834 (2016).

47 Krechmer, J. E., Pagonis, D., Ziemann, P. J. & Jimenez, J. L. L. Quantification of gas-wall partitioning in Teflon environmental chambers using rapid bursts of low-volatility oxidized species generated in-situ. *Environmental Science and Technology* **50**, 5757-5765 (2016).

48 Matsunaga, A. & Ziemann, P. J. Gas-wall partitioning of organic compounds in a Teflon film chamber and potential effects on reaction product and aerosol yield measurements. *Aerosol Science and Technology* **44**, 881-892 (2010).

49 Saleh, R., Donahue, N. M. & Robinson, A. L. Time scales for gas-particle partitioning equilibration of secondary organic aerosol formed from alpha-pinene ozonolysis. *Environmental Science and Technology* **47**, 5588-5594 (2013).

50 Donahue, N. M. *et al.* Critical factors determining the variation in SOA yields from terpene ozonolysis: A combined experimental and computational study. *Faraday Discussions* **130**, 295-309 (2005).

51 Offenberg, J. H., Kleindienst, T. E., Jaoui, M., Lewandowski, M. & Edney, E. O. Thermal properties of secondary organic aerosols. *Geophysical Research Letters* **33** (2006).

52 Stanier, C. O., Pathak, R. K. & Pandis, S. N. Measurements of the volatility of aerosols from α-pinene ozonolysis. *Environmental science & technology* **41**, 2756-2763 (2007).

53 May, A. A. *et al.* Gas-particle partitioning of primary organic aerosol emissions:(2) Diesel vehicles. *Environmental science & technology* **47**, 8288-8296 (2013).

54 Haddad, I. E. *et al.* Insights into the secondary fraction of the organic aerosol in a Mediterranean urban area: Marseille. *Atmospheric Chemistry and Physics* **11**, 2059-2079 (2011).

55 Haddad, I. E. *et al.* Primary sources of PM 2.5 organic aerosol in an industrial Mediterranean city, Marseille. *Atmospheric Chemistry and Physics* **11**, 2039-2058 (2011).

56 Minguillón, M. *et al.* Fossil versus contemporary sources of fine elemental and organic carbonaceous particulate matter during the DAURE campaign in Northeast Spain. *Atmospheric Chemistry and Physics* **11**, 12067-12084 (2011).

57 Crippa, M. *et al.* Wintertime aerosol chemical composition and source apportionment of the organic fraction in the metropolitan area of Paris. *Atmospheric Chemistry and Physics* **13**, 961-981 (2013).

58 Zotter, P. *et al.* Radiocarbon analysis of elemental and organic carbon in Switzerland during winter-smog episodes from 2008 to 2012–Part 1: Source apportionment and spatial variability. *Atmospheric Chemistry and Physics* **14**, 13551-13570 (2014).

59 Liu, S. *et al.* Secondary organic aerosol formation from fossil fuel sources contribute majority of summertime organic mass at Bakersfield. *Journal of Geophysical Research: Atmospheres* **117** (2012).
